# Homeostatic sleep regulation in the absence of the circadian sleep‐regulating component: effect of short light–dark cycles on sleep–wake stages and slow waves

**DOI:** 10.1186/s12868-021-00619-2

**Published:** 2021-02-27

**Authors:** Örs Szalontai, Attila Tóth, Máté Pethő, Dóra Keserű, Tünde Hajnik, László Détári

**Affiliations:** In vivo Electrophysiology Research Group, Department of Physiology and Neurobiology, Institute of Biology, Department of Physiology and Neurobiology, Eötvös Loránd University, Pázmány Péter sétány 1/C, 1117 Budapest, Hungary

**Keywords:** Short light-dark cycles, Homeostatic sleep regulation, Circadian sleep regulation, Slow waves, Sleep effect of light

## Abstract

**Background:**

Aside from the homeostatic and circadian components, light has itself an important, direct as well as indirect role in sleep regulation. Light exerts indirect sleep effect by modulating the circadian rhythms. Exposure to short light-dark cycle (LD 1:1, 1:1 h light - dark) eliminates the circadian sleep regulatory component but direct sleep effect of light could prevail. The aim of the present study was to examine the interaction between the light and the homeostatic influences regarding sleep regulation in a rat model.

**Methods:**

Spontaneous sleep–wake and homeostatic sleep regulation by sleep deprivation (SD) and analysis of slow waves (SW) were examined in Wistar rats exposed to LD1:1 condition using LD12:12 regime as control.

**Results:**

Slow wave sleep (SWS) and REM sleep were both enhanced, while wakefulness (W) was attenuated in LD1:1. SWS recovery after 6-h total SD was more intense in LD1:1 compared to LD12:12 and SWS compensation was augmented in the bright hours. Delta power increment during recovery was caused by the increase of SW number in both cases. More SW was seen during baseline in the second half of the day in LD1:1 and after SD compared to the LD12:12. Increase of SW number was greater in the bright hours compared to the dark ones after SD in LD1:1. Lights ON evoked immediate increase in W and decrease in both SWS and REM sleep during baseline LD1:1 condition, while these changes ceased after SD. Moreover, the initial decrease seen in SWS after lights ON, turned to an increase in the next 6-min bin and this increase was stronger after SD. These alterations were caused by the change of the epoch number in W, but not in case of SWS or REM sleep. Lights OFF did not alter sleep–wake times immediately, except W, which was increased by lights OFF after SD.

**Conclusions:**

Present results show the complex interaction between light and homeostatic sleep regulation in the absence of the circadian component and indicate the decoupling of SW from the homeostatic sleep drive in LD1:1 lighting condition.

## Background

Sleep is regulated by the complex interactions of homeostatic and circadian factors (Borbély et al. 2016; Deboer 2018). Homeostatic sleep regulation is demonstrated by the increased need for sleep after sleep deprivation (SD) (Borbely et al. 1981; Trachsel et al. 1986). Recovery sleep (RS) is characterized by an increase in the power of delta range (0.5–4 Hz) of the local field potentials (LFPs) during slow wave sleep (SWS) which is considered to be a measure of sleep intensity (Borbely and Achermann 1999). Slow wave activity (SWA) mostly reflects the rhythmic alternations of hyperpolarization (DOWN state) and depolarization (UP state) of cortical neurons (Vyazovskiy et al. 2011) called slow cortical rhythm (SCR) (Steriade et al. 1993a, b, c). Slow waves (SWs) are high amplitude, low frequency, synchronous LFP activity patterns representing the DOWN states of the SCR (Vyazovskiy et al. 2007b; Hajnik et al. 2013). The increment of SWA is the highest immediately after sleep deprivation (SD), mainly due to the higher incidence of slow waves represented by DOWN states (Vyazovskiy et al. 2009; Hajnik et al. 2013).

The circadian regulation of sleep is controlled by the suprachiasmatic nuclei (SCN). The activity of the SCN cells is modulated by the light information from the retina via mainly the retinohypothalamic tract (Moore and Lenn 1972). Visual information shaping the function of the SCN is also provided from the visual thalamus via the geniculohypothalamic tract (Card and Moore 1989). Daily rhythms such as S-W cycle, drinking, feeding, locomotor activity, body temperature, corticosterone level etc. are adjusted to the length and phase of the actual light-dark (LD) cycle (Stephenson et al. 2012). In most experiments, LD cycle is set to 12 hours of light and 12 hours of dark conditions (LD12:12). However, a variety of studies examined the effect of different lighting conditions on the rodent sleep–wake (S-W) patterns (Borbely et al. 1975; Alfoldi et al. 1991; Usui et al. 2003; Deboer et al. 2007; Vyazovskiy et al. 2007a; Hubbard et al. 2013). After several days under constant lighting conditions (constant light, constant dark) or in short light-dark cycle (LD 1:1, 1:1 h light - dark), the circadian rhythm free-runs with a different period of time depending on light intensity (Aschoff 1960). Several weeks in these conditions produce the complete disruption of circadian rhythm (Usui et al. 2003; Deboer et al. 2007).

Aside from the homeostatic and circadian components, light has itself an important, direct as well as indirect role in sleep regulation. Light exerts indirect sleep effect by modulating the circadian rhythms (Deboer 2018). Light also affects the S-W cycle directly as light pulses induce deep SWS in nocturnal animals as photic entrainment (Borbely 1976; Alfoldi et al. 1991). The direct effect is mediated via the track originating from the melanopsin-containing, intrinsically photosensitive ganglion cells of the retina (Lazzerini Ospri et al. 2017). Recent concepts identified the light as the third factor in the sleep regulation as the direct photic regulation is interacting with the circadian and homeostatic components to determine the timing and quality of the S-W cycle (Hubbard et al. 2013).

In the present study, rats were exposed to short light-dark pulses (LD1:1) which was found to eliminate circadian rhythm in behavior and motor activity (Borbely and Huston 1974) as well as in the S-W parameters (Borbely et al. 1975). Aside from the general characterization of S-W patterns during LD1:1 lighting condition, immediate S-W effects of light switches (both ON and OFF) were also examined. The short period light-dark cycling used in the present study allowed the examination of the homeostatic sleep regulation by SD in a condition lacking the circadian sleep-regulating component but allowed the light to express its direct regulatory role. As SWs represent the DOWN state of the SCR (Cash et al. 2009), analysis of SW parameters during both baseline LD1:1 condition and after SD provided novel information about the direct effect of light regarding the induction and maintenance of SCR in the absence of the circadian regulatory component. Understanding the effect of light on sleep stages and sleep regulation may bear tremendous importance in the resolution of several light-related human sleep problems evoked by long-term circadian disruption caused by shift-work, or environmental light pollution itself (Navara and Nelson 2007).

**Fig. 1:**
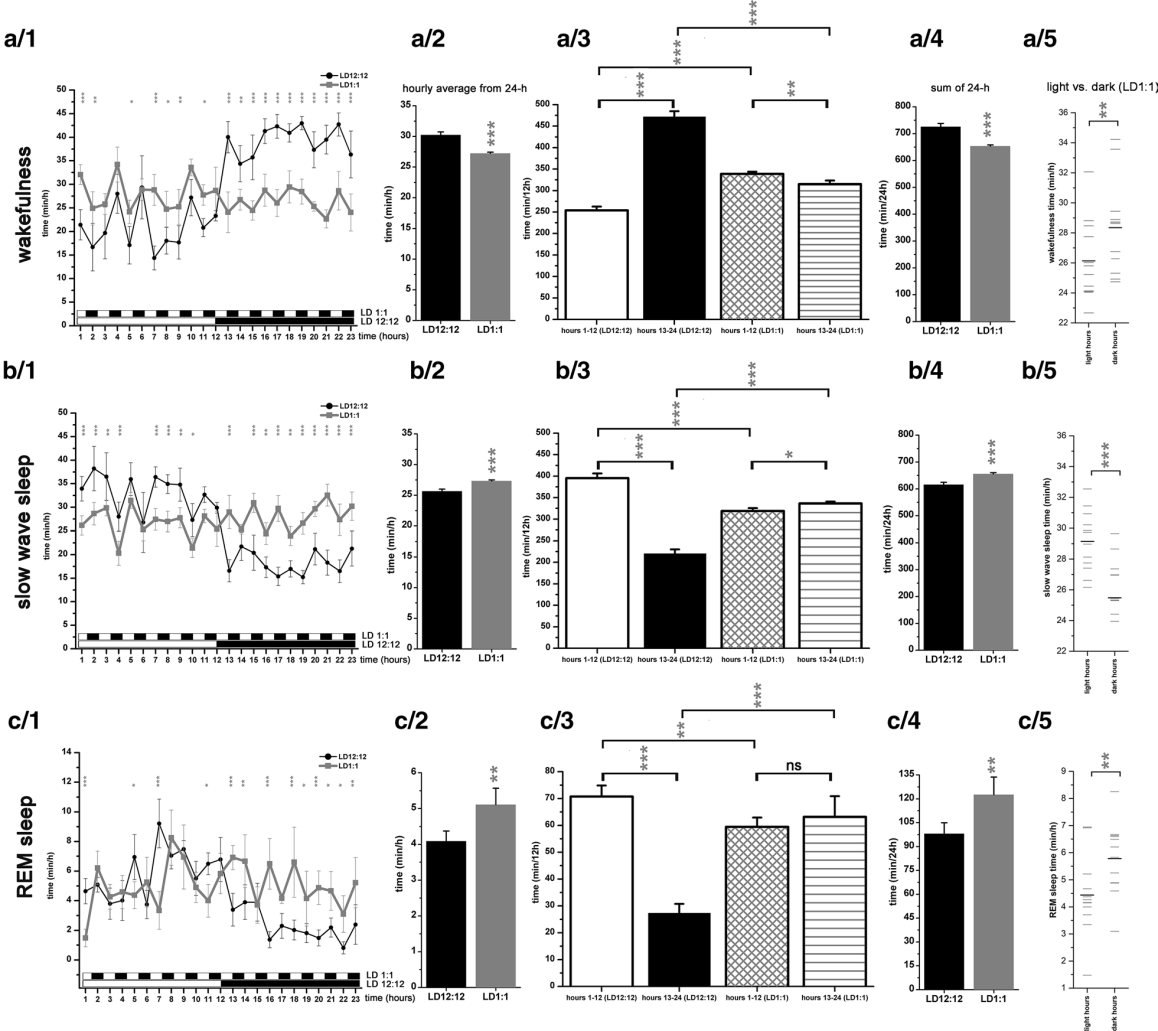
Sleep–wake parameters during baseline LD12:12 (n = 6) and baseline LD1:1 (n = 5) conditions. Line A: wakefulness, line B: slow wave sleep, line C: REM sleep. **a**/1, **b**/1, **c**/1: hourly averages of the times spent in the given vigilance stage. Thin black line: LD12:12; thick grey line: LD1:1. **a**/2, **b**/2, **c**/2: hourly averages of the times spent in the given vigilance stage calculated from the 24-h data in LD12:12- and in LD1:1 conditions. **a**/3, **b**/3, **c**/3: summarized time spent in the given vigilance stage during the first- and the second half of the day (CT1-CT12 hours and CT13-CT24 hours) in LD12:12- and in LD1:1 conditions. **a**/4, **b**/4, **c**/4: summarized time spent in the given vigilance stage during the whole day (24-h) in LD12:12- and LD1:1 conditions. **a**/5, **b**/5, **c**/5: hourly averages of the times spent in the given vigilance stage averaged separately for the light- (CT1, CT3, etc.) and for the dark hours (CT2, CT4, etc.) in LD1:1 condition. Black bars: average of the whole period (bright hours vs. dark hours); grey bars: average in the separate hours. SEM values are not depicted on panel **a**/5, **b**/5 and **c**/5. Black and white bars at the x axis of the panels **a**/1, **b**/1 and **c**/1 show the illumination pattern in the different hours. Grey asterisks (*) indicate significant deviation from the corresponding control (LD12:12) data (**a**/1, **b**/1, **c**/1) or indicate significance difference between the data derived from different time periods (LD12:12 baseline vs. LD1:1; **a**/2-**a**/4, **b**/2-**b**/4, **c**/2-**c**/4) or showing significant difference between the values belonging to light vs. dark hours (**a**/5, **b**/5, **c**/5). Significance was tested with two-way ANOVA with time and treatment as factors, followed by Sidak's multiple comparisons test. Sleep–wake times in light vs. dark hours in the LD1:1 condition depicted on **a**/5, **b**/5 and **c**/5 were compared using Welch’s t-test. Significance levels: *—p < 0.05; **—p < 0.01; ***—p < 0.001. Data are expressed as mean ± S.E.M

## Results

### General characterization of sleep‐wake stages during baseline in LD 12:12 and LD 1:1 conditions

The nocturnal nature of the laboratory rats was evident in the normal LD schedule (LD 12:12) when rats had less W in the LP (CT1-CT12 hours) and amount of both SWS and REM sleep were higher during the DP (CT13-CT24 hours; Fig. 1A1, B1 and C1). According to the Lomb-Scargle periodogram analysis (data not shown), the circadian rhythmicity was completely absent after at least 21 days spent in LD 1:1 condition. In LD 1:1, hourly average of W decreased (Welch’s t-test, p < 0.001; Fig. 1A2), while SWS and REM sleep increased (Welch’s t-test, p < 0.001 for SWS; p = 0.001 for REM sleep) (Fig. 1B2 and C2) compared to the LD 12:12 condition as control. In LD1:1 condition, all three S-W stages showed little difference between the CT1-CT12 period and the CT13-CT24 period corresponding to LP and DP in LD 12:12 control condition (Fig. 1A3, B3 and C3). The distribution both for W and SWS between the 1st and the 2nd half of the day was reversed in LD1:1 compared to LD12:12. W was significantly enhanced during CT1-CT12 period compared to the CT13-24 period (interaction; F (1, 18) = 896.4, p < 0.001; Fig. 1A3). SWS amount was significantly higher in the second half of the day (CT13-CT24) compared to the CT1-CT12 period (interaction; F (1, 18) = 680.5, p < 0.001; Fig. 1B3). The difference seen in REM sleep distribution favoring the CT1-CT12 period in LD12:12 was not present in LD1:1 where no difference was seen in the summarized REM sleep amount between CT1-CT12 vs. CT13-CT24 (Fig. 1C3).

**Fig. 2:**
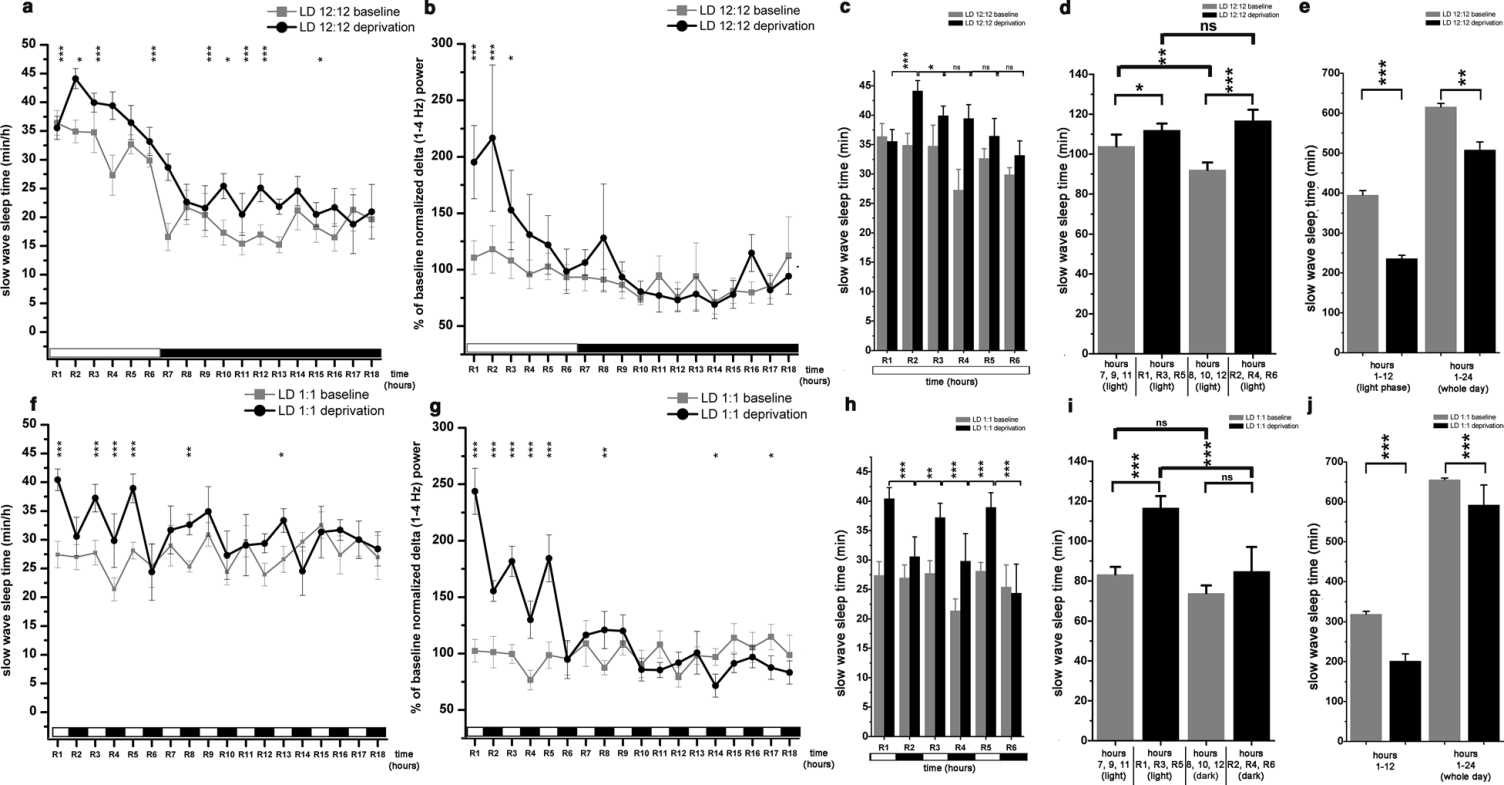
Effect of 6-h gentle handling total sleep deprivation on slow wave sleep and delta power during LD12:12 (n = 6) and LD1:1 (n = 5) conditions. The figure depicts only the data of the recovery period after SD (R1–R16 hours). **a–e**: LD12:12 data, panel F-J: LD1:1 data. **a**, **f**: Hourly average of slow wave sleep times during baseline (grey line) and after sleep deprivation (black line). **b** and **g**: Changes of normalized delta (1–4 Hz) power expressed as percentage of corresponding baseline. Grey lines: baseline; black lines: after SD. **c** and **h**: Slow wave sleep times (min/h) in the consecutive hours of the R1–R6 period. In LD12:12, the R1–R6 period consisted bright hours only (**c**) while in LD1:1, R1–R3-R5 hours were bright and R2-R4-R6 hours were dark (**h**). **d** and **i** Slow wave sleep times in blocks containing the sum of the times of three different hours taken from the baseline (hours 7, 9, 11 and hours 8, 10, 12) and from the recovery period (R1, R3, R5 and R2, R4, R6 hours). Panel **e** and **j**: summarized amount of slow wave sleep at the end of the light phase (hour CT12) during the baseline LD12:12 and LD1:1 conditions and after total SD (hour R6). The column pair at the right side show summarized slow wave sleep time at the end of the day during baseline and after total SD. Black and white bars at the x axis show the illumination pattern in the different hours. Significance was tested with two-way ANOVA with time and treatment as factors, followed by Sidak’s multiple comparisons test. Summarized SWS sleep times during the first- and in the second half of the day depicted on E and J were compared using Welch’s t-test. Significance levels: *, # - p < 0.05; **, ##- p < 0.01; ***, ### - p < 0.001. Data are expressed as mean ± S.E.M

12-hours sum of the LD 1:1 data significantly differed from the corresponding LD 12:12 control ones in all three vigilance stages (Fig. 1A3, B3 and C3). Interestingly, the distribution of the S-W stages between the first and the second half of the day was exactly the opposite in LD 1:1 as seen during the LD 12:12 cycles. More W but less SWS and REM sleep was seen during the first 12 hours in the LD 1:1 condition compared to the second half of the day (Fig. 1A3, B3, C3). Sum of 24-h data showed that the largest relative change was seen in the LD1:1 REM sleep compared to the LD 12:12 condition (25 %, p = 0.001 by Welch’s t-test; Fig. 1C4). Decrease of W and increase of SWS was smaller (9.9 % for W; 6.45 % for SWS) compared to the control LD 12:12 values, but both were also significant (p < 0.001 in both cases by Welch’s t-test) (Fig. 1A4 and B4). All three S-W stages showed uneven distribution between the light- and the dark hours in LD1:1. Both W and REM sleep were reduced in the bright hours compared to the dark ones (p = 0.001 for W, p = 0.008 for REM sleep; Welch’s t-test) (Fig. 1A5 and C5) while SWS was elevated in the bright hours (p < 0.001; Welch’s t-test) (Fig. 1B5).

### Total sleep deprivation in LD 12:12 and LD 1:1 conditions

#### Effect on slow wave sleep

During the SD sessions, no sleep did occur in any of the rats subjected to the 6-h gentle handling SD in any of the sessions. SD sessions always started at the same time in a bright hour. Intensity of rebound sleep was characterized by delta power enhancement after the SD when sleep was allowed again. For this reason, delta power values were analyzed hourly and power values seen during the SD period as well as during the corresponding baseline recordings were not plotted on Fig. 2.

During LD 12:12 and LD 1:1 condition, intense recovery occurred in the first third of the recovery period (R1–R6 hours) (Fig. 2a and f). Recovery continued with lower intensity until the end of the recording day between R7-R18 hours. In both cases, the recovery was not complete even at the end of the day, as SWS amount was significantly lower compared to the baseline amount period (interaction; F (1, 16) = 5.132, p = 0.04 for LD1:1; Fig. 2j) (interaction; F (1, 20) = 24.10, p < 0.001; Fig. 2e). The relative recovery was stronger in LD 1:1 condition as sleep debt at the end of the day was lower compared to the LD 12:12 condition (9.6 % vs. 17.5 %) (Fig. 2e vs. j).

**Fig. 3:**
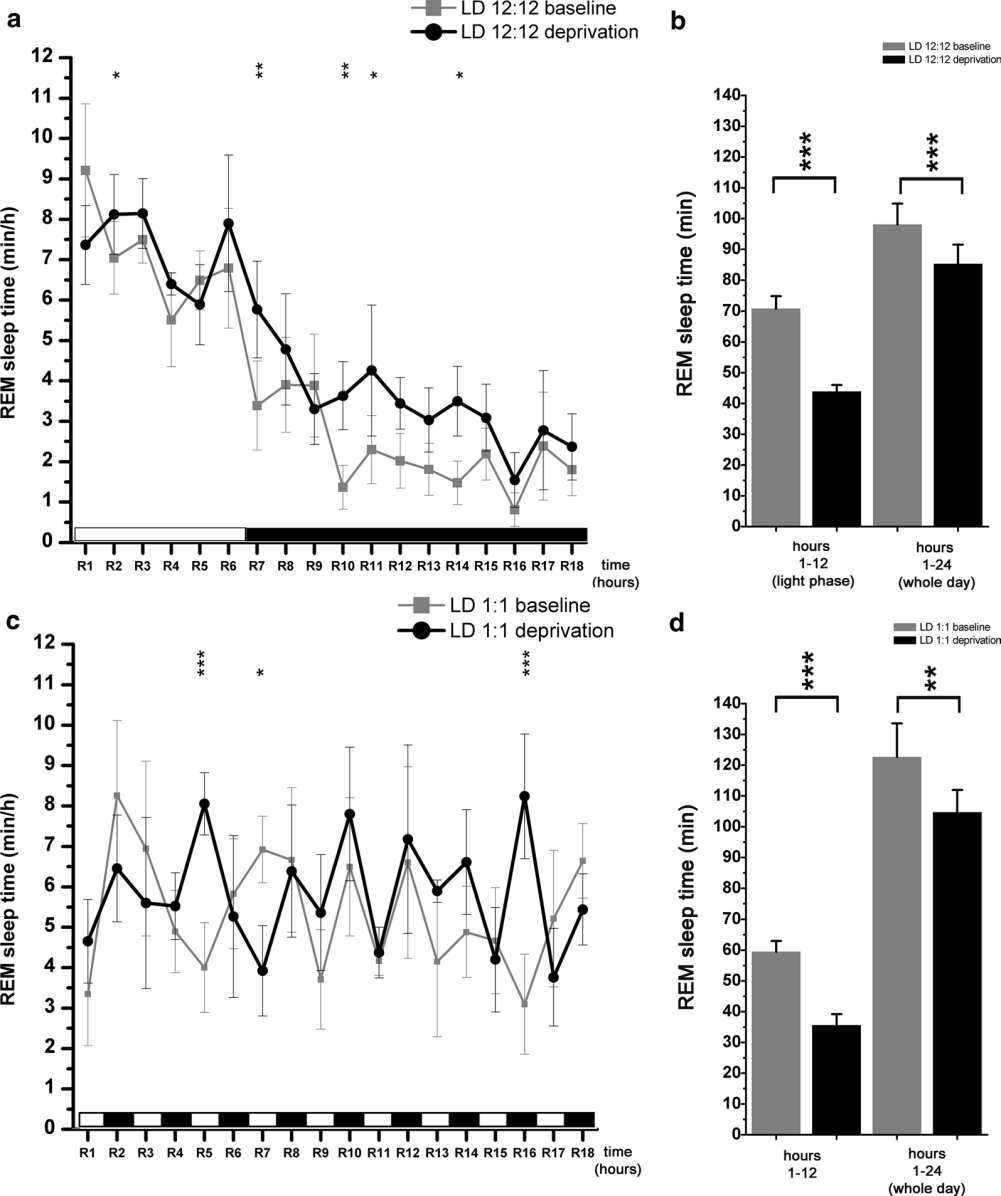
Effect of 6-h gentle handling total sleep deprivation on REM sleep during LD12:12 (n = 6) and LD1:1 (n = 5) conditions. The figure depicts only the data of the recovery period after SD (R1–R16 hours). Panel **a**–**b**: LD12:12 data, panel **c**–**d**: LD1:1 data. Panel **a** and **c**: hourly average of REM sleep times during baseline (grey line) and after sleep deprivation (black line). Panel **b** and **d**: summarized amount of REM sleep at the end of the light phase (hour CT12) during the baseline LD12:12 and LD1:1 conditions and after total SD (hour R6). The column pair at the right side show summarized REM sleep time at the end of the day during baseline and after total SD. Black and white bars at the x axis show the illumination pattern in the different hours. Significance was tested with two-way ANOVA with time and treatment as factors, followed by Sidak’s multiple comparisons test. Summarized REM sleep times during the first- and in the second half of the day depicted on C and D were compared using Welch’s t-test. Significance levels: *, # - p < 0.05; **, ##- p < 0.01; ***, ### - p < 0.001. Data are expressed as mean ± S.E.M

In LD 1:1, sleep replacement was more intense in the bright hours in the R1–R6 period analyzed. Pair-wise comparison of the consecutive hours in the R1–R6 hours showed significant fluctuation of the SWS amount displaying higher values during the bright hours (Fig. 2h). Similar comparison in LD 12:12 showed no such pattern as recovery peaked in the R2 hour then declined in the consecutive hours (Fig. 2c). Summarized SWS amount in the R1, R3, R5 hours (light) was significantly higher compared to the dark hours of this period (R2, R4, R6) (interaction; F (5, 60) = 9.028, p < 0.001; Fig. 2I). The same comparison in LD 12:12 showed no significant difference (Fig. 2d).

Increased intensity characteristic to RS mirrored in the delta power increment in the recovery period (Fig. 2b and g). Delta power was significantly increased in the R1–R3 period in LD 12:12 compared to baseline (interaction; F (17, 180) = 6.622, p < 0.001; Fig. 2b), while all hours in the R1–R5 period showed significant delta power elevation in LD 1:1 compared to baseline (interaction; F (17, 144) = 33.59, p < 0.001; Fig. 2g). Delta power increment was larger in the bright hours (R1, R3, R5) reflecting more intense and longer SWS epochs in the bright hours compared to the dark ones (Fig. 2i). Temporal dynamics of the increased delta power was similar to that seen for the SWS in both lighting conditions. In LD 12:12, R2 hour showed the strongest increase, then a decline was seen. In LD 1:1, strongest delta power elevation was seen in the R1 hour then, delta power fluctuated according to the bright or dark nature of the actual hour.

### Effect on REM sleep

#### LD12:12 condition

After the 6-h total REM sleep deprivation, REM sleep replacement did not occur as a definitive rebound in the 2nd half of the LP when sleep was possible again neither in the LD12:12 condition (Fig. 3a) nor in the LD1:1 condition (Fig. 3c). Instead, REM sleep replacement was seen mainly in the DP in LD12:12. Significant amount of REM sleep debt was present at the end of the LP (R6 hour) as well as at the end of the day (interaction; F (1, 20) = 11.24, p < 0.001 for both the LP and for the whole day replacement; Fig. 3b).

**Fig. 4:**
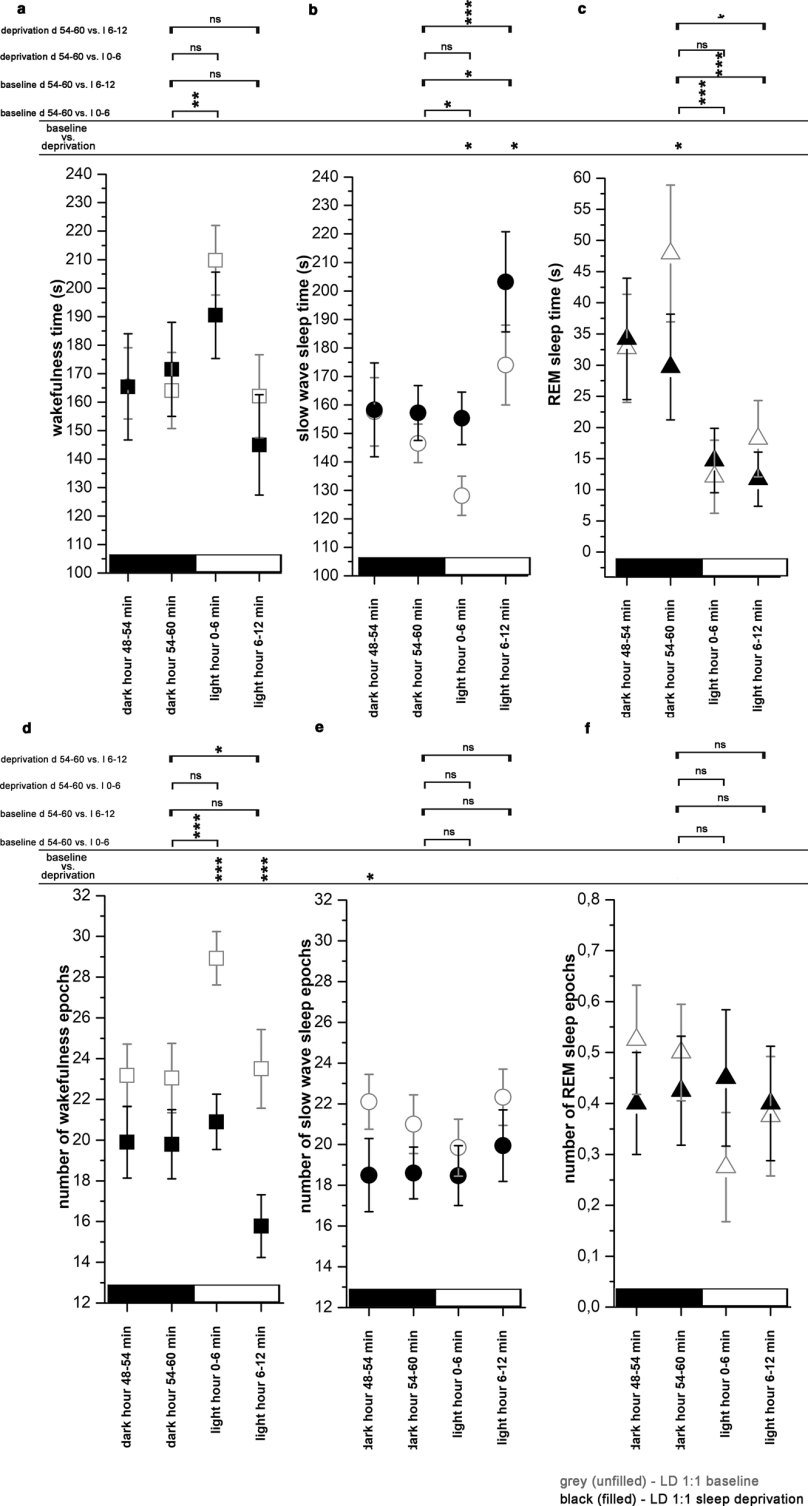
Effects of light onsets regarding S-W times (**a**-**c**) and epoch numbers (**d**-**f**) in LD1:1 (n = 5) condition. S-W data were averaged in consecutive 6-min long bins using the data of all light switches during the 24-h recordings. Two bins were analyzed in the pre-switch period (dark hour 48–54 min and 54–60 min) and two bins for the post-switch period (bright hour 0–6 min and 6–12 min). Panel **a** and **d**: wakefulness; panel **b** and **e**: slow wave sleep; panel **c** and **f**: REM sleep. Significance was tested with two-way ANOVA with time and treatment as factors, followed by Sidak’s multiple comparisons test. Differences were checked between the different 6-min time bins before and after the light switch during baseline and after SD and between the baseline vs. after SD values regarding the same time period. Significance levels: * - p < 0.05; ** - p < 0.01; *** - p < 0.001. Data are expressed as mean ± S.E.M

#### LD1:1 condition

In contrast to the SWS replacement, no systematic relationship was seen between the light- or dark nature of the hour and the amount of REM sleep replenished during the recovery period in LD1:1 after sleep deprivation (Fig. 3c). Similar to the REM sleep replacement in the LD12:12 condition, significant REM sleep debt was found both at the end of the LP (R6 hour) and the end of the recording day (R18 hour) (treatment; F (1, 16) = 44.09, p < 0.001 for the LP replacement, p = 0.002 for the whole day replacement; Fig. 3d). Relative sleep debt at the end of the day was slightly larger in LD1:1 condition compared to the LD12:12 one (14,6 % vs. 13 %).

### Effects of light switches on sleep–wakes stages in LD1:1 condition

As light changes can evoke immediate S-W effects, effects of light switches from OFF to ON, and ON to OFF were analyzed in 6-min bins following the changes.

#### Effect of lights ON – baseline

Lights ON during the baseline significantly increased W- and decreased SWS- and REM sleep time (interaction; F (6, 48) = 36.05, p < 0.001 for both the W and for the REM sleep, p = 0.05 for SWS) (Fig. 4a–c). Elevation of W time was due to the increase of the number of W epochs (interaction; F (6, 48) = 21.98, p < 0.001) (Fig. 4d) while number of SWS epochs and REM sleep epochs was not changed (Fig. 4f). After the temporary decrease following the light switch, SWS time became significantly elevated in the second post-switch bin compared to the second pre-switch bin value (interaction; F (6, 48) = 36.05, p < 0.001) (Fig. 4b) but this elevation was not accompanied by the change of the SWS epoch number (Fig. 4e).

**Fig. 5:**
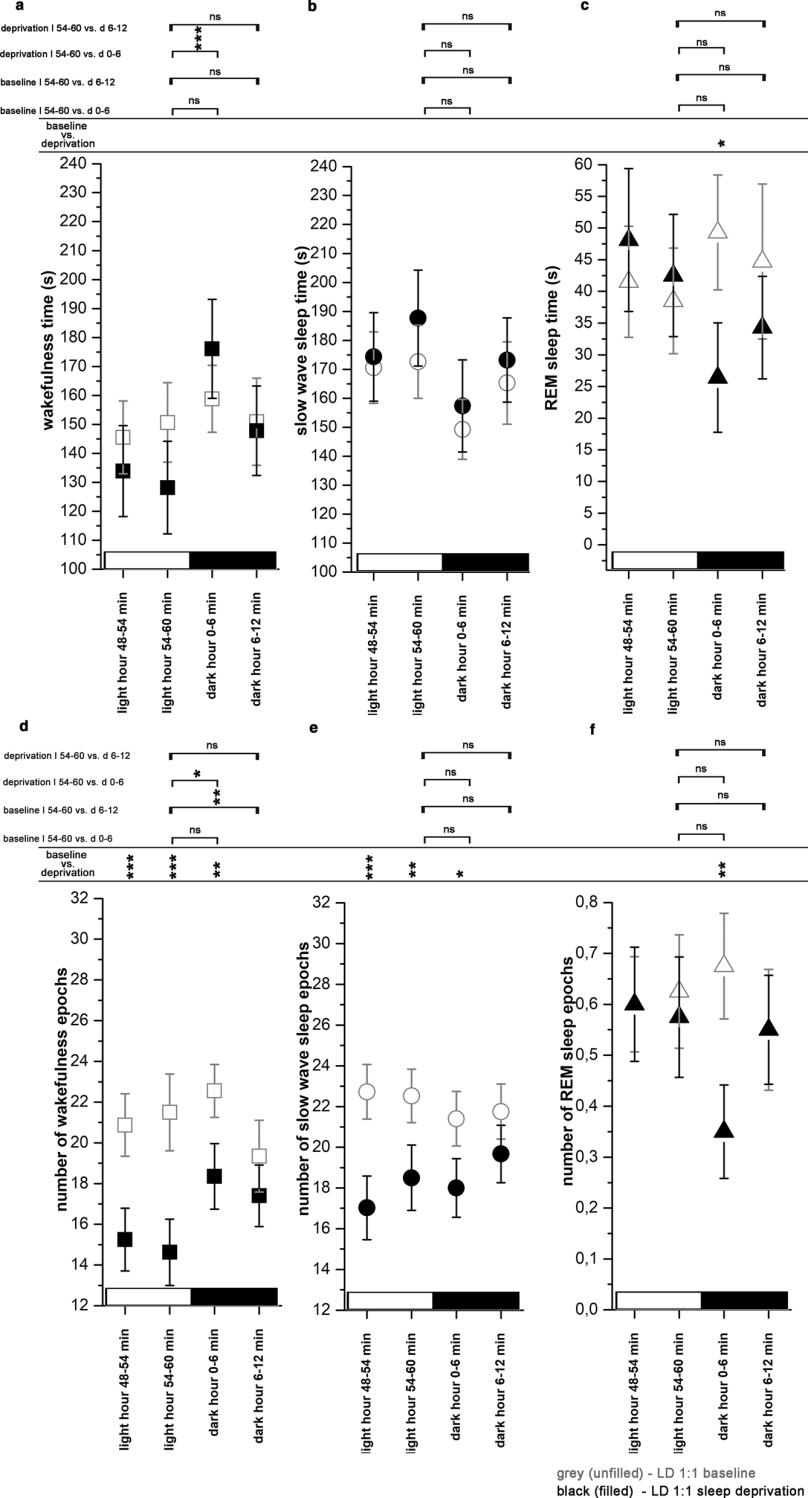
Effects of dark onsets regarding S-W times (**a**–**c**) and epoch numbers (**d**–**f**) in LD1:1 (n = 5) condition. S-W data were averaged in consecutive 6-min long bins using the data of all light switches during the 24-h recordings. Two bins were analyzed in the pre-switch period (dark hour 48–54 min and 54–60 min) and two bins for the post-switch period (bright hour 0–6 min and 6–12 min). **a** and **d**: wakefulness; panel **b** and **e**: slow wave sleep; **c** and **f**: REM sleep. Significance was tested with two-way ANOVA with time and treatment as factors, followed by Sidak's multiple comparisons test. Differences were checked between the different 6-min time bins before and after the light switch during baseline and after SD and between the baseline vs. after SD values regarding the same time period. Significance levels: *—p < 0.05; **—p < 0.01; ***—p < 0.001. Data are expressed as mean ± S.E.M

#### Effect of lights ON – after total SD

After total SD, light switch did not cause immediate changes neither in the amount of the vigilance stages (Fig. 4a–c) nor in the epoch number of the stages (Fig. 4d–f). However, SWS elevation was significant in the second post-switch bin (bright hour 6–12 min) compared to the second pre-switch bin (treatment, F (3, 32) = 17.06, p < 0.001) (Fig. 4b). SWS elevation in this period was significantly greater compared to that seen in the baseline (treatment, F (3, 32) = 17.06, p = 0.02). Similarly to the baseline, SWS changes after SD evoked by the light switch were not caused by changes in SWS epoch numbers (Fig. 4e).

REM sleep time decreased in the second post-switch period compared to the second pre-switch period after total SD (interaction, F (3, 32) = 3.840, p = 0.02) (Fig. 4c). The relative drop of the REM sleep amount after the lights ON was larger after the SD compared to the baseline, as REM sleep time was significantly higher in the second pre-switch bin after SD compared to the baseline (interaction, F (3, 32) = 3.840, p = 0.02) (Fig. 4c).

#### Effects of lights OFF- baseline

Generally, S-W effects of lights OFF were much weaker compared to the effects of lights ON. Light OFF did not cause immediate changes neither in the amount of the vigilance stages (Fig. 5a–c), nor in the epoch number of the stages during the baseline (Fig. 5a–f), and no effects were seen in the second post-switch period as well.

**Fig. 6:**
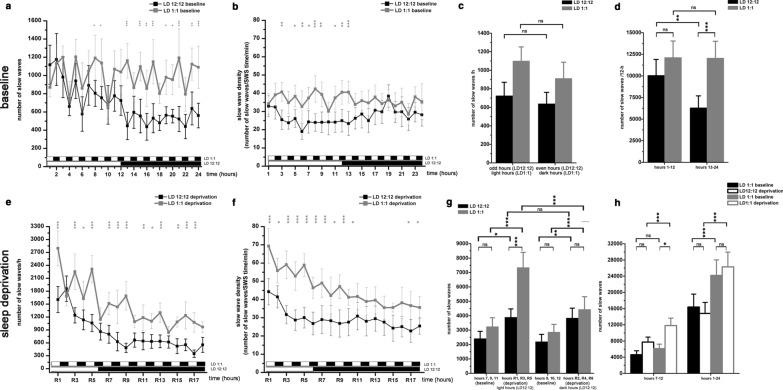
Number of slow waves during baseline and after SD during LD12:12 (n = 6) and LD1:1 (n = 5) conditions. Top row: baseline data, bottom row: sleep deprivation data. **a** and **e**: hourly number of SWs during baseline and after SD in LD1:1 and LD12:12 conditions. **b** and **f**: slow wave density (number of SWs/SWS time/min) during baseline and after SD in LD1:1 and LD12:12 conditions. Panel **c**: averaged SW numbers/hour in odd and even hours (LD12:12) and in light and dark hours (LD1:1). **d**: SW numbers/12 h in the CT1-CT12 and CT13-CT24 periods, in LD1:1 and LD12:12 conditions. **g**: SW numbers in the first third of the recovery period (R1–R6 hours) SW numbers during CT9, CT11, CT13 baseline hours vs recovery period hours R1, R3, R5 (bright hours in LD1:1). SW numbers during CT8, CT10, CT12 baseline hours vs recovery period hours R2, R4, R6 (dark hours in LD1:1). **h**: SW numbers summarized in different time periods after SD compared to the corresponding baseline periods without SD. Black and white bars at the x axis show the illumination pattern in the different hours. Significance was tested with two-way ANOVA with time and treatment as factors, followed by Sidak's multiple comparisons test. Significance levels: *, #—p < 0.05; **, ##- p < 0.01; ***, ###—p < 0.001. Data are expressed as mean ± S.E.M

#### Effect of lights OFF – after SD

In contrast to the baseline, dark switch significantly increased W time (treatment, F (3, 32) = 3.242, p < 0.001) (Fig. 5a) and W epoch number (treatment, F (3, 32) = 5.149, p = 0.02) after total SD (Fig. 5d) while SWS and REM sleep were not affected (Fig. 5b–f). Number of W- and SWS epochs were significantly lower in both pre-switch periods and the first post-switch period (Fig. 5d and e). Number of REM sleep epochs was also lower after SD in the first post-switch period compared to the baseline (interaction, F (3, 32) = 5.269, p = 0.001) (Fig. 5f).

### Slow waves

#### Changes of slow wave parameters in baseline LD 12:12 and LD 1:1 conditions

**Fig. 7:**
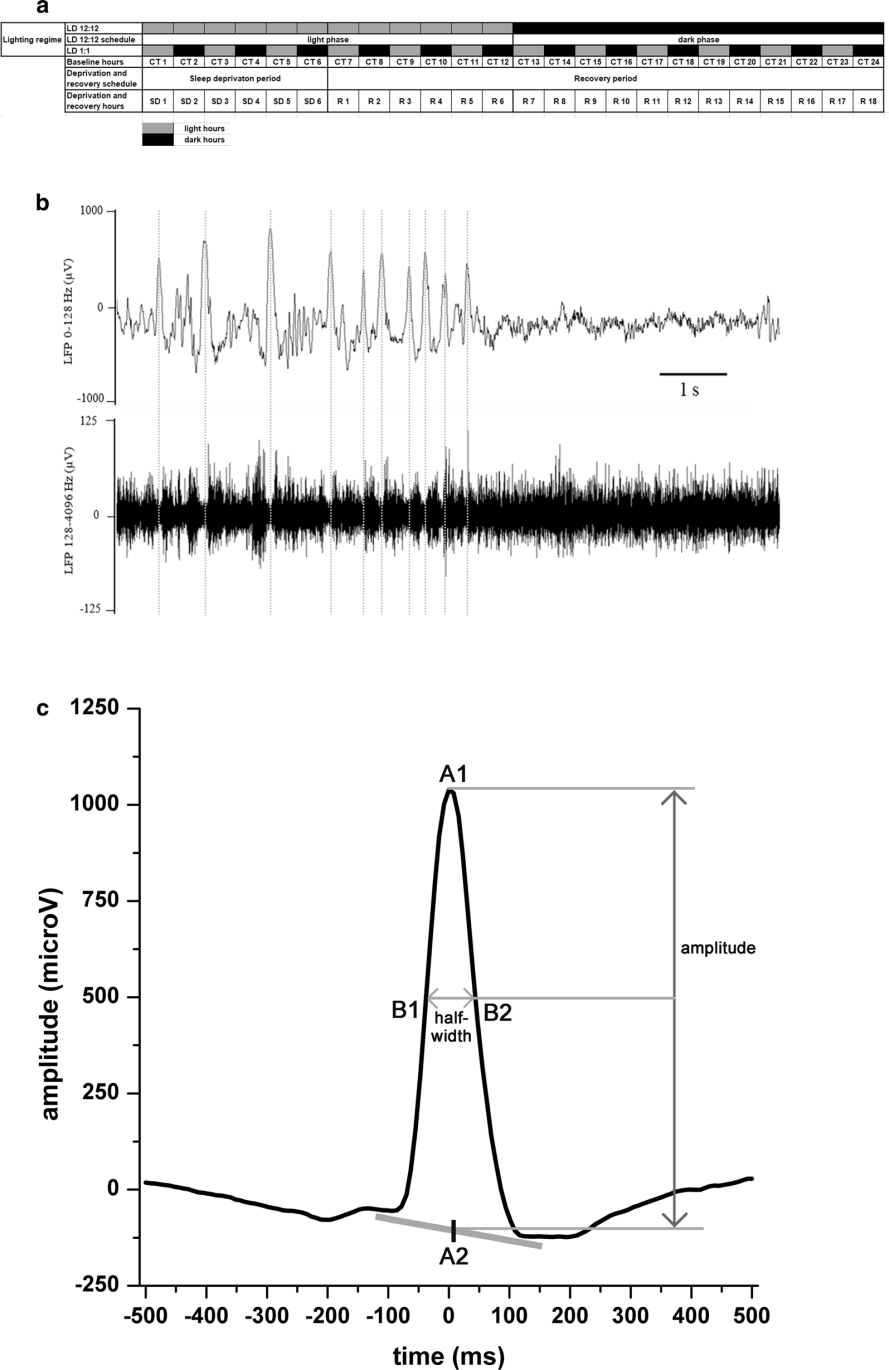
**a**: Overview of the lighting conditions and the nomenclature used for the different hours and longer time periods used by this study. Baseline hours named as CT1–CT24 for both lighting conditions (LD12:12 and LD1:1). 6-h total sleep deprivation hours are referenced as SD1–SD6 while the subsequent recovery period is consisted of hours R1–R18. **b** 10-s representative LFP sample data (low-pass filtered at 128 Hz, top row) from the R1 hour after SD performed in LD12:12 condition. Bottom row shows the corresponding raw multiple unit data filtered between 128 Hz and 4096 Hz. Vertical dotted lines indicate the maxima of the LFP slow waves, the latter represent the DOWN states of the ongoing SCR. On the multiple unit data, the cease of firing is clearly visible simultaneously with the presence of the slow waves. **c** Parameters of the slow waves analyzed. After the extraction of the slow waves from the raw LFP data, slow waves were averaged for ± 500 ms around the maxima in 1-h long time bins. Then amplitude (A2–A1 in μV), half width (B2–B1 in ms) and slow wave number were summarized in 1-h bins

Among the SW parameters analyzed in this study (number, amplitude and halfwidth; see Fig. 7c), only the number showed significant changes during the different recording conditions. Thus, amplitude and halfwidth data are not shown. Because of this, only the analysis regarding the SW numbers is presented here. SW density (number of SWs/SWS time/min) was calculated to characterize the intensity of SW-generating mechanisms during SWS.

During baseline conditions, number of slow waves was significantly higher in the LD1:1 condition compared to the LD12:12 one (interaction, F (23, 216) = 3.859, p < 0.001) (Fig. 6a). LD1:1 SW numbers were lower only in the CT1-CT2 period compared to the LD12:12 data, due to the highest homeostatic sleep pressure seen at the beginning of the LP in LD12:12 condition. Significantly higher SW numbers in LD1:1 were seen mainly during the second half of the day (CT13-CT24 hours), due to the reduced amount of SWS in the DP in LD12:12 condition (Fig. 6a). No significant difference was seen in SW numbers neither between odd- and even hours (LD12:12) nor between light- and dark hours (LD1:1) (Fig. 6c). In LD12:12 condition, number of SWs was significantly lower during the DP (interaction, F (1, 18) = 5.998, p < 0.01) (Fig. 6d), due to the reduced amount of SWS characteristic for this period (see Fig. 1B3). In contrast, no difference was seen in the SW number between the CT1-12 period and CT13-24 period in LD1:1 condition (Fig. 6d). SW number was significantly higher in the CT13-24 period in LD1:1 condition compared to the LD12:12 condition (interaction, F (1, 18) = 5.998, p < 0.001) while there was no difference in the CT1-12 period using the same comparison (Fig. 6d).

SW density was significantly higher in LD1:1 condition compared to the LD12:12 condition (treatment, F (1, 216) = 139.8, p < 0.001). Significant elevation was seen mainly in the first half of the day (Fig. 6b).

#### Changes of slow wave variables after SD in LD 12:12 and LD 1:1 conditions

Both in LD 12:12 and LD 1:1 conditions, SD caused strong increase in the number of SWs in the recovery period compared to the baseline without SD (Fig. 6e). Generally, more SWs were present during the recovery period in the LD1:1 condition compared to the LD12:12 one (Fig. 6e). The difference was significant in the R1–R6 period but also for the whole day of the SD session (interaction, F (3, 36) = 9.237, p < 0.001 for both comparisons) (Fig. 6e). Even if the recovery of the SWS after SD was not complete in sleep time neither in LD12:12 nor in LD1:1 (Fig. 2e and j), numerically the lost SWs were completely replenished in both lighting conditions (Fig. 6h right columns).

The general elevation seen in the number of the SWs after SD was more pronounced in the bright hours in the R1–R6 period in the LD1:1 condition as the total number of SWs was significantly larger in the R1–R3-R5 period (bright hours) compared to the R2-R4-R6 period (dark hours) (interaction, F (1, 20) = 18.00, p < 0.001). Similar correlation was not seen in the LD12:12 data (Fig. 6g).

SW density was significantly higher in the LD1:1 condition compared to the LD12:12 condition (treatment, F (1, 162) = 273.1, p < 0.001). Significant elevation was seen mainly in the first half of the recovery period (R1–R11 hours) (Fig. 6f).

## Discussion

In the present work, homeostatic sleep regulation and spontaneous S-W stages were examined in rats exposed to short light-dark cycles (LD1:1), when circadian sleep regulation was absent, but direct sleep effect of light could prevail. Our novel results reflect the intriguing relationship between effect of light and the homeostatic sleep regulation examined by SD during LD1:1 condition.

### Sleep–wake stages in control LD 12:12 and LD 1:1 conditions

In the normal light-dark schedule, nocturnal animals sleep more in the LP, and are more awake in the DP (Borbely and Neuhaus 1978; Clancy et al. 1978). During LL (Depres-Brummer et al. 1995) or DD (Stephan 1983) conditions, the circadian rhythm of the S-W cycle becomes free-running. The same free-running rhythm could be observed after 1–2 weeks in short light-dark (LD 1:1) conditions. After 2 or more weeks in this lighting regime, the circadian rhythm of the S-W cycle became completely abolished (Usui et al. 2003), as it was also seen in the present study. In our experiments, animals kept in LD 12:12 slept significantly more SWS and REM sleep in the LP and had more W during the DP, in agreement with previous reports (Borbely and Neuhaus 1978; Clancy et al. 1978).

The present results regarding baseline LD1:1 S-W parameters are in good agreement with previous experimental data showing general SWS and REM sleep facilitation and W depression in similar lighting conditions both in rats (Borbely et al. 1975) and in mice (Deboer et al. 2007). The facilitating effect of light on SWS was found to be proportional with the intensity of the light pulses (Borbely et al. 1975). However, light intensity was kept constant throughout the present experiments and the effect of different light intensities on sleep was not examined.

The facilitating effect of light on REM sleep in LD1:1 condition was considered to be secondary to the increase in SWS (Borbely et al. 1975), as REM sleep strongly depends on SWS. The presence of some SWS is a prerequisite for the development of REM sleep. During the sleep-dominated LP, the two sleep stages have a spontaneous cycle with a period time of about 12–20 minutes in rat in LD12:12 condition (Trachsel et al. 1991). Indeed, when the direct effect on lights ON was examined on a finer (6-min) time scale, SWS and REM sleep both decreased temporarily immediately after the lightning change, then both stages were enhanced in the next 6-min period, in agreement with previous data (Borbely et al. 1975; Borbely 1980).

REM sleep episodes were shifted to the dark hours in agreement with previous results (Alfoldi et al. 1991) showing the effect of the lights OFF (Borbely 1976; Deboer et al. 2007). However, the immediate REM sleep-inducing effect of dark onset was not seen in our data, as REM sleep time only non-significantly increased in the next 6-min bin after the dark onset compared to the pre-switch bin. The REM sleep-inducing effect of dark onset could depend on the ongoing sleep phase of the rat (Tsai 2005), and the occurrence of REM sleep epochs is not frequent. Thus, REM sleep epoch might not be induced in the 6-min periods analyzed here, if the dark onset found the rat in W and not in SWS which is a prerequisite for REM sleep induction. The occurrence of REM sleep is a function of a certain amount of preceding SWS (Usui et al. 2003). The shorter light periods promoted the timing of SWS to the bright hours. However, the occurrence of REM sleep episodes shifted - at least, partly- to the dark hours as the bright hour had been terminated when a certain amount of REM sleep episodes could be expressed. Number of REM sleep epochs was not changed at around the light switches showing that changes of the environmental lighting do not affect the REM sleep inducing mechanisms directly but, may alter the REM sleep maintenance mechanisms as seen in the change of the length of the REM sleep episodes. Taken together, SWS and REM sleep are regulated by the light on separate ways.

Another factor may contribute in the REM sleep enhancement seen in baseline LD1:1 condition. Non-natural lighting conditions and light itself were considered to be an aversive factor for nocturnal animals despite these animals prefer dark conditions to sleep (Fishman and Roffwarg 1970; van Betteray et al. 1991). Short light-dark cycles may evoke slight distress in the rats which was found to be associated with enhanced REM sleep in case of several stressful situations as acute immobilization stress (Hegde et al. 2011), controllable or escapable footshock (Sanford et al. 2010) and acute food deprivation (Jacobs and McGinty 1971).

Light has an immediate, short-term awakening effect in rodents (Pilorz et al. 2016), similarly to the dark onset (Altimus et al. 2008; Lupi et al. 2008). This effect was evident in the present experiments, and this effect might have interfered with the direct SWS-inducing effect of the light as the latter effect was delayed after the light onset because of the sensory and behavioral activation evoked by the change of the environmental illumination itself.

### Homeostatic sleep regulation during LD1:1 lighting condition

SWS recovery after 6-h total SD in LD1:1 condition was more intense both in time and in the increase of the delta power compared to LD12:12. SWS replacement during both conditions followed the same temporal dynamics. SWS recovery was the most intense immediately after the SD period in hours R1–R6. This finding indicates that homeostatic sleep need can evolve even in the absence of circadian influences. SWS-inducing direct effect of the light shaped the temporal dynamics of the sleep replacement in the recovery period that is primarily governed by the exponential dissipation of the homeostatic sleep drive according to the classic view of the two-process sleep regulation model (Borbely 1982). The shaping effect of light caused that SWS compensation was more intense in the bright hours both in time and in the augmentation of the delta power. Taken together, the connection between the homeostatic component and the light seems to be synergistic in the bright hours. However, during the dark hours, W-inducing effect of the darkness temporarily blocked the dissipation of the homeostatic sleep drive. Delta power increment in the recovery period was based on the increase of the SW number in both lighting conditions in agreement with our previous data showing that the increase of the SW number is the main source of the LFP delta power enhancement seen during RS in LD12:12 condition (Hajnik et al. 2013). As the number of SWs reflect sleep intensity (Vyazovskiy et al. 2011; Hajnik et al. 2013) and SW number was elevated during the baseline conditions in LD1:1 compared to LD12:12, SD caused more loss from an already more intense sleep in LD1:1. The intensity of the RS was above of the baseline sleep as reflected in the SW density of the SWS in the recovery period.

### Homeostatic influences vs. effect of the light

High homeostatic sleep pressure can overcome the temporary W-inducing and SWS-suppressing effect of light onset as these effects were not visible after the SD. These results are in good agreement with recent concepts emphasizing the role of the light in the sleep regulation interacting with both the circadian- and the homeostatic component to achieve the best timing and quality for the sleep (Hubbard et al. 2013). In the absence of the circadian regulation seen in LD1:1 condition, light could have more impact on the homeostatic regulation (Borbély et al. 2016).

SWs were strongly increased in the first hour of the recovery period (R1). As 6-h SD was applied, the first recovery hour was in the light period according to the scheme used in both LD1:1 and LD12:12 conditions. In this case, not only the increased homeostatic need for sleep was mirrored in the strong elevation of the SW number but also the direct effect of light. The latter effect is responsible for the SW number elevation in later bright hours during the recovery period (most prominent hours were R3 and R5) while SW numbers during the dark hours of the recovery period failed to exceed significantly the values seen in the same period after LD12:12 SD.

Short light-dark cycles may have an impact in the homeostatic regulation itself, as they might prevent the buildup of the normal homeostatic sleep pressure. W induced in the dark hours is opposed in the next bright hour, when the SWS-inducing effect of light can be expressed. This is reflected in the general decrease of the W amount during the whole day. Thus, LD1:1 lighting condition represents a state, when not only the circadian regulation is eliminated but the homeostatic regulation is also deteriorated at least in a short-term way.

In the LD12:12 lighting condition, increased delta power and increased SW density reflects the increased homeostatic sleep drive. That is evident at the beginning of the light phase following the night, during which W is more prevalent, and the homeostatic sleep drive is accumulating. However, increased SW density during the bright hours in LD1:1 condition reflects the direct SWS- and SW-inducing effect of the light and not the homeostatic sleep pressure, as alternating light-dark cycles prevent the long-term accumulation of the homeostatic sleep pressure. Thus, LD1:1 condition may represent a state when the homeostatic sleep regulation and SWs are dissociated from each other. Similar decoupling was found using the chronic sleep restriction paradigm (Stephenson et al. 2015). According to this hypothesis, SWs are not the indicators of recovery processes as well as the hoemostatic sleep pressure in LD1:1 but reflecting the direct sleep-inducing effect of light instead.

## Conclusions

Present results show the complex interaction between light and homeostatic sleep regulation in the absence of the circadian component and indicate the decoupling of SW from the homeostatic sleep drive in LD1:1 lighting condition demonstrated firstly by the present study. Taken together, the LD1:1 lighting regime combined with SD can gain importance as a tool for studying the interaction of the direct sleep effect of light and homeostatic regulation, as light-induced sleep problems become more and more frequent in the human population worldwide (Cho et al. 2015; Xu and Lang 2018; Blume et al. 2019).

## Methods

### Surgery

Male Wistar rats (n = 6, weighing between 300 and 330 g at the time of surgery) were anesthetized with ketamine/xylazine (80 mg/kg ketamine and 10 mg/kg xylazine, i.p.) and fixed in a stereotaxic frame (David-Kopf). Bipolar concentric electrodes (125 µm polyimide insulated stainless steel wire in a 23G stainless steel tube) were implanted to the vertex (Br: -4.5 mm, L: 2.0 mm). The wire reached the layer V 1.2 mm below while the tube had contact with the dura mater. Stainless steel screws (0.8 mm, Fine Science Tools, USA) were placed above the frontal sinus and cerebellum for reference and grounding purposes. To record EMG activity, a pair of 250 µm diameter, teflon-insulated stainless steel wires (California Fine Wire, CA, USA) were inserted into the neck musculature, close to the skull. All wires were soldered to a miniature female connector that was fixed to the skull with cranioplastic cement (PlasticsOne Inc, VA, USA). During surgery, body temperature was maintained at 37 °C by a heating pad (Supertech Ltd., Pecs, Hungary). Recording sessions started after 1–2 weeks of recovery. The surgery and electrode implantation was similar to that published earlier by our laboratory (Hajnik et al. 2013). Following surgery, the animals were kept warm, and painkillers (50 mg/kg metamizole, i.p.) was administered for 3 days.

Experiments were carried out in accordance with the Hungarian Act of Animal Care and Experimentation (1998, XXVIII) and with the directive 2010/63/EU of the European Parliament and of the Council of 22 September 2010 on the protection of animals used for scientific purposes. Experimental protocols were approved by the Ethical Board of Eötvös Loránd University. Efforts were made to minimize the number of animals used.

### Housing

Rats were kept in LD 12:12 cycle (light-dark 12 h: 12 h, 21 days) then transferred to LD 1:1 (light-dark 1 h: 1 h) lighting condition for another 21 days. Recordings started at 9:00 AM (bright hour). The intensity of lighting during bright hours was ∼ 90–100 lux. All animals were housed individually in Plexiglas cylinders (height: 345 mm, diameter: 350 mm). Cages were located in a sound-attenuated room with controlled ambient temperature of 21 °C. Water and standard laboratory chow (ToxiCoop Ltd., Budapest) were available *ad libitum*. Rats were connected to the recording system through flexible, zig-zag flat cables attached to fixed swivels (Plastic One or Litton) above the home cages. This arrangement provided free movements for the rats. Cables were connected to the rats three days before the recordings started to let the animals habituate to the cables. Experimental setup was similar to that previously described (Bertram et al. 1997; Tóth et al. 2007; Hajnik et al. 2013; Borbely et al. 2018).

### Timeline and experimental conditions

Experiments started by a control recording of spontaneous S-W activity for 24 hours (CT1-CT24 hours) both for LD12:12 and LD1:1 conditions. SD sessions lasted for 6 hours followed by an 18-h recovery period. Four experimental conditions were defined: LD 12:12 – baseline, LD 12:12 – SD and recovery, LD 1:1 – baseline and LD 1:1 – SD and recovery (Fig. 7). LD1:1 condition data were compared to LD12:12 condition data as control.

### Electrophysiological recordings

LFP was measured through home-designed headstages based on the TLC2264I amplifiers (Texas Instruments, USA) built into the male connector. Signals were amplified and filtered (500X, 0.3 Hz – 4 kHz, Elsoft Bt.) then digitalized and saved by an analog–to–digital converter (A/D) card (National Instruments, Austin, TX, USA, LabView). Sampling rate was set to 8192 Hz (a power of 2) to facilitate fast Fourier transformation. Original recordings were downsampled to 128 Hz to yield 512 data points per 4-sec recording time for LFP analysis. All LFP and EMG data obtained during the recording sessions were stored on hard disk for offline analysis.

### Sleep deprivation

6-h total SD was performed using the gentle handle method. Whenever the animals appeared drowsy or slow waves emerged in the LFP, the animals were mildly disturbed by moderate noise, fragrant soap, cardboard pieces, bedding materials from other animal or by introducing fresh water or fresh food. During the gentle handling method, the animals were never touched (Franken et al. 1991).

### Sleep scoring

Sleep stages were scored using custom-made semi-automatic software. The program enabled visual inspection of the recorded signals, digital filtering, and spectral analysis of the LFP curves. Power spectra were calculated using the FFT algorithm for all consecutive 4-s periods from all recordings. Power was integrated in the delta (0.5-4 Hz), theta (4–10 Hz), alpha (10–14 Hz), beta (14–30 Hz) and gamma (30–48 Hz) frequency ranges and the ratio of the theta/delta power determined. EMG data was also processed using the FFT method and the total power (variance) was calculated in the 5–48 Hz range.

Epochs containing movement artifacts (high delta power and high EMG variance) and rapid eye movement (REM) sleep epochs (low delta power, high theta/delta ratio, and low muscle tone) were manually selected in all recordings by visual inspection of the LFP and EMG signals.

Epochs containing artifacts were excluded from further analysis (less than 1 % of the baseline recording and recovery and around 5 % during SD).

There are several scoring methods to distinguish SWS and W, either automatic, based on calculation of sophisticated variables from the recorded data (Robert et al. 1999), or manually, relying on the decision of an experienced scorer visually inspecting the LFP, EMG and power curves (Neckelmann et al. 1994). In all cases, slow wave (< 4 Hz) content of the LFP and the level of EMG activity are the most important indicators used as delta power changes are closely and inversely related with the level of cortical arousal (Trachsel et al. 1989). That semi-automated scoring method used here had been published earlier (Détári et al. 1993) and was used in several studies published by our laboratory later (Détári et al. 1999; Tóth et al. 2007; Toth et al. 2012; Hajnik et al. 2013, 2016; Borbely et al. 2018).

In the present experiments, delta power and EMG thresholds were set individually for each rat by visually inspecting of the raw LFP and EMG data from control recordings. These objective thresholds were then used to score recordings obtained after the treatments. Epochs in which delta power was above and EMG value below these thresholds were marked as SWS, while epochs with lower delta power or higher EMG activity as W. Raw hypnograms were smoothed, i.e. every 32-s period was assigned to the dominant sleep-W stage (Figueroa Helland et al. 2010).

For the analysis of the effects of light switches, S-W epochs were classified in every 4 s-long time bins. In this case, smoothing of the hypnogram for longer time periods (i.e. 32 s) was not applied as the aim was to analyze S-W transitions around the light switches using a finer time scale. S-W data were average in consecutive 6-min long bins using the data of all light switches during the 24-h recordings. Two bins were analyzed in the pre-switch period (dark hour 48–54 min and 54–60 min) and two bins for the post-switch period (bright hour 0–6 min and 6–12 min).

### Analysis of rebound sleep

To examine the effect of the total SD on sleep and the following rebound, amounts of SWS and REM sleep were analyzed in different time periods. Normalized delta power was also calculated in the same periods. As rebound sleep was expected to be the most pronounced in the first 6 hours after the end of the SD, these hours (R1–R6) were analyzed separately. Sleep replacement was separately analyzed in the bright and in the dark hours of this period in LD1:1 condition. In some cases, the first, intense rebound seen in the R1–R6 hours was not able to completely compensate for the lost sleep and delta power. To get a better picture about the long-term recovery process, S-W stages and delta power changes were followed until the end of the recovery period (R18 hour).

### LFP power analysis

LFP power values were analyzed independently from the vigilance stages. Power values in the delta (1–4 Hz), theta (4–10 Hz), alpha (10–16 Hz), beta (16–30 Hz) and gamma (30–48 Hz) frequency bands independently from the vigilance levels (W, SWS, REM sleep) were averaged for 60-minute long periods and normalized using the data of the defined baseline day which was used as reference for the SD and recovery recordings. Grand average of the power values of all hours and frequency bands of the baseline day was calculated as ’normalization factor’. Then each of the power values belonging to any time point were divided by the same ’normalization factor’ in case of both the baseline and the treatment recordings. Normalized values then summarized for 60-minutes long periods. After that, values of the 1-h periods of the baseline day were averaged separately in the different bands. Actual values of the 1-h periods were divided by this daily average and expressed as average % by frequency bands. Normalized values belonging to the SD days were also divided by the baseline daily average separately for each frequency bands and expressed as baseline %.

Only the data belonging to the delta range presented here. Data from the 0–1 Hz LFP range were completely excluded from the analysis as artifacts originating from cable movements fell mostly in this range.

### Slow wave selection and analysis

To select large, deep-positive LFP waves, a preset threshold was determined for each rat separately. The threshold was set manually displaying compressed view of the LFP signal showing W epochs belonging to the LP on computer monitor. An artefact-free, 16 s-long LFP epoch was selected and the value being numerically twice as large as the highest amplitude value seen during this epoch was used to specify the limit for slow waves (SW). LFP intervals around the peak of SWs (± 500 msec) were selected, counted and averaged for each consecutive 60-minutes and 6-minutes periods. The amplitude of the averaged SW was defined as the difference between the maximal positive value and the mean of the two local minima around the peak. Duration was calculated as the width at half amplitude of the waves. Thus, incidence, amplitude and duration values of the SWs were analyzed as previously described by our laboratory (Hajnik et al. 2013) (Fig. 7b and c).

### Histology

After recordings were completed, the experimental animals were euthanized. Rats were deeply anaesthetized with urethane (1.2 g/kg i.p.). Animals were perfused transcardially with 150 ml physiological saline followed by 500 ml 4 % phosphate-buffered paraformaldehyde. After perfusion, brains were removed from the skull, and postfixed in the same solution overnight, at 4 °C. 50 µm coronal slices were cut by a vibratome, mounted onto gelatine-coated slides and stained with gallocyanine for 24 hours followed by covering with Depex. Cortical recording sites were located with a light microscope (Leica DM2500, camera: Olympus DP73) using the stereotaxic atlas Paxinos and Watson (1998) as reference.

### Statistical analysis

Sleep-W parameters (time, epoch number and epoch length) and normalized LFP power values expressed in baseline% were analyzed statistically by two-way ANOVA with time and treatment as factors, followed by Sidak’s multiple comparisons test. In case of the SD analysis, SWS and REM sleep values were also summarized for different time periods including sums based upon the different illumination (light vs. dark hours) in case of the SD day and the baseline day, in both lighting conditions. Similar comparisons were made regarding the SW numbers. In these cases, sleep data were compared by Welch’s t-test. In each series of experiments, homogeneity of variances and normal distribution of data was tested before statistical analysis. All tests were two-tailed and p < 0.05 was accepted as the lowest limit of significant difference. Data are shown as mean ± S.E.M. on figures. Statistical analysis was performed using Prism 7.0 (GraphPad Software, San Diego, USA). Data were plotted in Microcal Origin 8.0 (OriginLab Corporation, Northampton, USA). Final editing was performed using Adobe Photoshop CC. Lomb-Scargle periodogram (Ruf 1999) was used to test the presence of the circadian rhythmicity in S-W stages.

## Data Availability

The datasets during and/or analysed during the current study available from the corresponding author on reasonable request.

## Abbreviations

DD: Constant dark
DP: Dark phase
EMG: Electromyogram
LD: Light–dark
LD 1:1: 1:1 h light–dark cycle
LD 12:12: 12:12 h light–dark cycle
LFP: Local field potential
LP: Light phase
R1–R6: First six hours of recovery after sleep deprivation
REM sleep: Rapid Eye Movement sleep
RS: Recovery sleep
SD: Sleep deprivation
S-W: Sleep–wake
SW: Slow waves
SWS: Slow wave sleep
W: Wakefulness
